# The Onset of Creutzfeldt–Jakob Disease After COVID-19: Navigating the Diagnostic Challenges and Clinical Evolution

**DOI:** 10.1155/crnm/6617766

**Published:** 2025-10-23

**Authors:** Ahmet Z. Burakgazi

**Affiliations:** ^1^Neurology Department, Southcoast Health, Fall River, Massachusetts, USA; ^2^Department of Neuroscience, Brown University, Providence, Rhode Island, USA

## Abstract

This case report presents a 47-year-old female who developed Creutzfeldt–Jakob disease (CJD) in a patient who had recently experienced a COVID-19 infection. The patient initially experienced speech changes and cognitive decline approximately 4-5 weeks after recovering from COVID-19. Over the course of several months, her condition rapidly deteriorated, progressing to severe neurocognitive decline, including catatonia, aggression, and delusions. Diagnostic findings included cortical ribboning on MRI, generalized periodic discharges on EEG, and elevated tau and 14-3-3 proteins with positive RT-QuIC in CSF analysis. Despite various treatments, including methylprednisolone and IVIG, the patient's condition worsened, leading to hospice care within a year of symptom onset. This case illustrates the diagnostic challenges in distinguishing prion diseases from other postviral complications, particularly when presuming a parainfectious inflammatory process without supporting evidence, which may delay definitive testing such as RT-QuIC. No causal relationship between COVID-19 and CJD can be established from this single case. This report underlines the need for further research into potential post-COVID-19 neurological complications.

## 1. Introduction

Since its emergence in late 2019, severe acute respiratory syndrome coronavirus 2 (SARS-CoV-2), the virus responsible for coronavirus disease 2019 (COVID-19), has posed a significant global health challenge. Beyond respiratory symptoms, COVID-19 has been associated with a wide array of neurological manifestations, such as altered mental status, encephalopathy, encephalitis, meningitis, postinfectious demyelination, headaches, dizziness, acute ischemic strokes, acute necrotizing hemorrhagic encephalopathy, cerebral venous sinus thrombosis, seizures, small fiber neuropathy, Guillain–Barré syndrome, critical illness polyneuropathy and myopathy, and olfactory dysfunction [1–3].

Creutzfeldt–Jakob disease (CJD) is one of the prion diseases, a group of disorders caused by the accumulation of misfolded prion proteins (PrPSc), which induce a cascade of neurodegenerative changes in the central nervous system [1]. The clinical presentation of CJD is often insidious but becomes rapidly progressive, with symptoms typically emerging in the sixth decade of life, although cases have been reported across all ages. The hallmark symptoms include rapid cognitive decline, myoclonus, visual disturbances, and ataxia [2]. In some instances, psychiatric symptoms such as depression, anxiety, and delusions may precede or coincide with neurological signs [3]. The disease typically progresses within 6 months to 1 year after symptom onset, with death occurring within a few months of diagnosis in most cases [1, 3, 4]. The potential connection between COVID-19 and CJD remains controversial [5]. While a few cases [4–6] in the literature suggest a temporal link between these two clinical entities, further research is needed to establish any causal relationship. The diagnosis of CJD is challenging, requiring vigilant clinical scrutiny for proper diagnosis. This case report describes a presentation of CJD in a patient who recently experienced a COVID-19 infection, emphasizing the diagnostic complexities when distinguishing prion disease from other postviral complications. This case report adds to the limited literature by describing the occurrence of CJD symptoms in close proximity to a prior COVID-19 infection, without implying causation, and highlights the importance of adhering to standard diagnostic protocols for rapidly progressive dementia, including early consideration of real-time quaking-induced conversion (RT-QuIC) testing in cases with characteristic magnetic resonance imaging (MRI) findings.

## 2. Case Presentation

**Figure 1:**
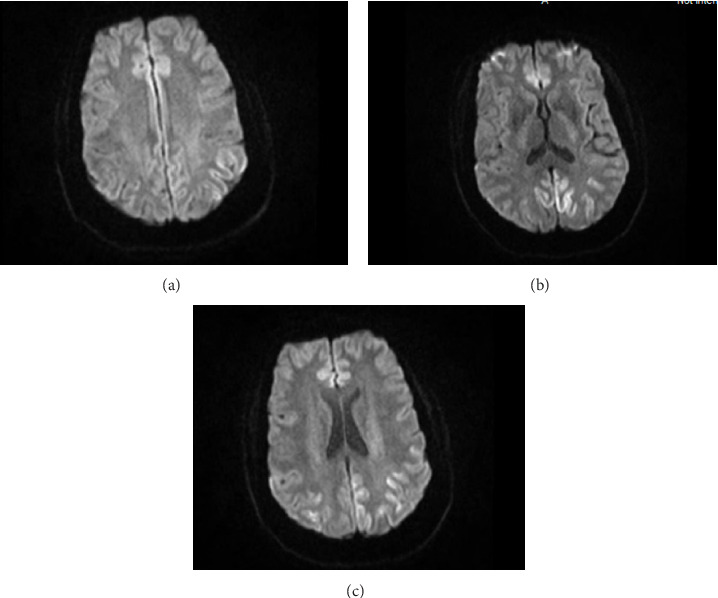
Brain MRI showing diffusion restriction in the bilateral occipital, parietal, and frontal lobes. (a) Diffusion-weighted imaging (DWI) demonstrating hyperintensity in the bilateral occipital lobes indicative of restricted diffusion. (b) DWI showing similar findings in the parietal lobes. (c) DWI revealing involvement of the frontal lobes.

A 47-year-old female with a history of hypertension was diagnosed with COVID-19 in October 2022, initially presenting with a dry cough, fatigue, shortness of breath, and exertion. She was treated with antibiotics and supportive care, and her COVID-19 symptoms resolved within 7–10 days. However, approximately 4-5 weeks later, in late November to early December 2022, she developed new-onset speech difficulties, including word-finding issues, stuttering, and signs of foreign accent syndrome. In January 2023, she underwent a brain MRI, which revealed diffusion restriction in the bilateral occipital, parietal, and frontal lobes (Figure 1). The initial electroencephalogram (EEG) findings were within normal limits. Following this, the patient was referred to the neurology department for further evaluation.

When she was seen by neurology personnel, her speech problem was more prominent. She had trouble with her speech, and she spoke with what was perceived by both the speaker and listeners as a Russian accent. She was an American native and did not previously possess this accent. She did not have any familial connection with Russia. The patient underwent lumbar puncture and electroencephalography, both of which were unremarkable.

During the first visit, the patient was placed on clonazepam (0.5 mg twice daily) and gabapentin (300 mg three times daily) for symptomatic management of speech difficulties and possible anxiety/neuropathic components. She returned to our clinic 6 weeks later for a follow-up visit, and no improvements were shown in her clinical symptoms. Due to abnormal MRI findings and possible inflammatory process, she was initially treated with methlyprednisolone infusion of 1 g per day for 5 days. However, she has yet to show any improvements in her clinical symptoms. The repeated MRI of the brain with and without the constant showed similar findings with no contrast enhancement. The patient subsequently returned to our clinic 3 months after the methylprednisolone infusion. No improvements were shown in her symptoms, and she was therefore treated with an intravenous immune globulin (IVIG) infusion of 1 g per kg per day for 2 days, every 4 weeks for 3 months. She returned to our clinic for a 3-month follow-up visit after completion of three sessions of IVIGs as well as a follow-up MRI of the brain with and without contrast. Given the ineffectiveness of the IVIG and the absence of changes in the clinical and MRI findings, the IVIG was discontinued. During the last visit (around 10 months after the initial presentation), the patient still had a significant speech problem. In May 2023, the patient was hospitalized for progressive neurocognitive decline, including catatonia, aggression, and delusions. MRI of the brain with and without contrast on May 12, 2023, showed progression of cortical diffusion restriction, now involving the temporal lobes in addition to the frontal, parietal, and occipital lobes. These changes were concerning for CJD, though initially interpreted in the context of possible progressive encephalitis. However, the autoimmune encephalitis panel was negative. A broad diagnostic workup, including lumbar puncture and EEG, was performed. CSF analysis revealed elevated tau protein (4500 pg/mL; reference range < 500 pg/mL), positive 14-3-3 protein, normal glucose (60 mg/dL; reference range 40–70 mg/dL), normal protein (35 mg/dL; reference range 15–45 mg/dL), and no pleocytosis (white blood cell count 2/μL; reference range 0–5/μL). An RT-QuIC test, initially negative, returned positive on June 2, 2023. Elevated tau protein and positive 14-3-3 protein are supportive of a CJD diagnosis even with a negative RT-QuIC.

Continuous EEG (cEEG) monitoring for over 1 week revealed significant findings of variable frequency generalized periodic discharges (GPDs) and ictal-interictal continuum (IIC) patterns, without clear visualization of definitive seizure activity. The MRI demonstrated cortical ribboning and gyriform hyperintensity in multiple brain regions, which, along with the positive RT-QuIC and the elevated tau and 14-3-3 proteins, was consistent with a diagnosis of CJD.

Despite symptomatic treatment with Depakote for behavioral control, the patient's condition continued to worsen. After a thorough discussion with the family and given the progressive deterioration in neurological function and the diagnosis of CJD, the decision was made to transition the patient to hospice care in June 2023. She was ultimately transferred to hospice on July 15, 2023, following a rapid decline in cognitive, speech, and physical function. The final diagnosis of CJD was confirmed based on clinical presentation, MRI findings, EEG findings, CSF analysis, and positive RT-QuIC testing. The patient passed away in September 2023. The sequence of events is summarized in the timeline presented in Table 1.

## 3. Discussion

This case report describes a complex clinical scenario of CJD following COVID-19 infection. CJD is a sporadic, rare, rapidly progressive, and fatal neurodegeneration disorder caused by the misfolding of the prion protein [2]. Possible temporal occurrence with SARS-CoV-2 infections has been observed in some reports, but this should not be interpreted as evidence of a causal relationship with prion-related neurodegenerative processes, though no evidence supports this [5]. SARS-CoV-2 may contribute to neurodegeneration by causing elevated levels of proinflammatory cytokines such as interleukin-6 (IL-6), tumor necrosis factor-alpha (TNF-α), and interleukin-1 beta (IL-1β), which can cross the blood–brain barrier and induce neuroinflammation [4, 5, 7]. COVID-19-related excessive inflammatory response may activate microglia and astrocytes that may exacerbate neuronal injury and promote prion protein misfolding [4, 5, 8]. It has been postulated that SARS-CoV-2 may directly influence protein aggregation [5]. The spike protein of SARS-CoV-2 contains prion-like domains that may facilitate interactions with amyloid-forming proteins, including PrP, tau, and alpha-synuclein [4, 5]. These interactions could promote prion-like propagation and accelerate neurodegenerative processes. With a similar pathophysiological interaction between COVID-19-related inflammation and prion-related protein misfolding may have occurred in our case, potentially acting as a trigger for pre-existing subclinical prion disease or accelerating the disease course [4, 5]. Therefore, the patient demonstrated rapid clinical progression of symptoms, from initial speech changes to severe rapid decline within months. A few similar cases of CJD in patients with a recent history of COVID-19 have been reported in the literature [4-6]. This case describes the onset of CJD in temporal proximity to a COVID-19 infection, but no causal relationship can be inferred. Hypotheses regarding potential mechanisms, such as neuroinflammation or prion-like domains in the SARS-CoV-2 spike protein, require further investigation.

CJD is a fatal degenerative brain disorder that is characterized by rapidly progressive dementia that leads to memory loss, personality changes, involuntary movements, and myoclonus along with various several psychiatric symptoms [3]. Most patients with CJD die within a year of diagnosis due to the rapid progression of the clinical picture [2]. EEG in CJD may show a generalized periodic sharp wave pattern [1], and CSF analysis may show elevated 14-3-3 protein [9]. In our case, the patient initially presented with memory impairment, behavior and personality changes, and speech disturbances. Speech changes, such as aphasia and dysarthria, are rare as the primary or isolated presenting symptom in sporadic CJD cases (estimated ∼1%), though they may occur more frequently (∼50% or higher) during the course of the illness, indicating that this onset presentation, while uncommon, is not unprecedented [10]. Over time, her condition deteriorated rapidly, with progressive neurocognitive decline that included catatonia, aggression, and delusions. Tragically, she passed away almost within 1 year of disease onset. Pertinent diagnostic findings in this case included positive diffusion restriction in the bilateral occipital, parietal, and frontal lobes on MRI of the brain; positive variable frequency GPDs and IIC patterns in EEG; and positive RT-QuIC [11] and elevated tau and 14-3-3 proteins in CSF analysis. CJD remains a diagnostic and therapeutic challenge due to its rapid progression and the lack of curative treatments [12]. Early recognition of its clinical and diagnostic features is crucial for providing supportive care and facilitating timely discussion with patients and their families about the prognosis.

The differential diagnosis for rapidly progressive dementia in the context of a recent COVID-19 infection is broad and complicated [2, 7]. Although COVID-19-associated encephalopathy should be considered, it is essential to maintain a careful consideration for other neurodegenerative disorders, including CJD [2]. Other potential diagnoses to be considered include autoimmune encephalitis, paraneoplastic syndromes, neurotoxic disorders, and rapidly progressive variants of more common neurodegenerative diseases such as Alzheimer's disease or dementia with Lewy bodies [2, 7, 9]. Vascular dementia, especially with involvement of the striatum, cortex, or thalamus, can also present with imaging findings similar to those of CJD. Additionally, infectious causes such as viral encephalitis, metabolic imbalances, and psychiatric conditions must be ruled out. Distinguishing CJD from its mimics often relies on clinical presentation, neuroimaging, and key findings such as myoclonus, visual disturbances, and MRI characteristics such as cortical ribboning and basal ganglia hyperintensities on diffusion-weighted imaging (DWI) [7, 13]. Finally, a definitive diagnosis of CJD typically requires a combination of clinical findings, neuroimaging, electroencephalography (EEG), and cerebrospinal fluid biomarkers, including 14-3-3 protein, tau protein, and the RT-QuIC assay [4, 11, 12]. In retrospect, given the rapidly progressive dementia and characteristic cortical ribboning on DWI MRI, RT-QuIC analysis should have been performed much earlier in the evaluation to expedite diagnosis.

EEG plays an important role in the diagnosis of CJD, with the findings evolving as the disease progresses [1]. In the early stage, EEG may show normal patterns or nonspecific changes including diffuse slowing or frontal intermittent rhythmic delta activity [1]. As CJD advances, more characteristic patterns may be seen, particularly periodic sharp wave complexes (PSWCs), which are detected in about two-thirds of sporadic CJD cases and typically appear 8–12 weeks into the disease course. PSWCs may initially be lateralized, then become generalized with 66%–68% sensitivity in probable CJD cases. It is important to realize that a normal or nonspecific EEG does not rule out CJD, particularly in early stages. This was evident in our case, where the EEG was within normal limits at the early stage of the disease. Then, repeated EEG in the later stage of the disease showed variable frequency of GPDs with IIC patterns, reflecting the evolving nature of CJD's electrophysiological manifestations [2, 4, 7, 12].

MRI of the brain provides crucial diagnostic clues in the diagnosis of CJD. DWI is the most sensitive sequence for identifying CJD-related findings [4, 13]. The hallmark MRI DWI finding is cortical ribboning, which presents as a hyperintense signal in the cerebral cortex affecting multiple lobes on both sides but often asymmetrically. Other CJD-related MRI findings include hyperintense signals in the caudate nuclei and putamina, thalamic involvement (pulvinar sign), combined involvement of the striatum and thalamus (hockey stick sign), and rapidly progressive cerebral atrophy [9, 13]. In our case, the MRI demonstrated cortical ribboning and gyriform hyperintensity in multiple brain regions, which is the most common and specific MRI findings of CJD. Notably, MRI findings, particularly DWI sequences have a reported sensitivity of over 90% for diagnosing CJD, making MRI an invaluable tool in the diagnostic work-up of suspected CJD cases [7, 13].

This case underscores the current limitations in CJD treatment, as neither immunomodulatory therapies nor symptomatic treatments significantly altered the disease course [1, 7]. In the context of a potential underlying inflammatory process, the patient underwent empirical administration of methylprednisolone and IVIG. The ineffectiveness of methylprednisolone and IVIG highlights the urgent need for novel therapeutic approaches in prion diseases. Looking ahead, this case report emphasizes several critical areas for future research and clinical practice [2, 8]. Longitudinal studies of neurological outcomes in COVID-19 survivors are essential, as is enhanced surveillance for rare neurodegenerative conditions in post-COVID-19 patients [12, 14]. Further investigation into potential mechanisms linking viral infections to prion diseases is crucial, as is the development of more sensitive and specific early diagnostic tools for CJD [2, 8].

Although several case reports have documented the onset of CJD in patients who had recently experienced COVID-19 infection, large-scale surveillance has found no evidence of increased CJD incidence or altered survival rates since the beginning of the COVID-19 pandemic [15–17]. While this case does not establish a causal link between COVID-19 and CJD, it illuminates the complex interplay between viral infections and neurodegenerative processes [12, 14]. The report underscores the importance of comprehensive neurological evaluations in patients with atypical post-COVID-19 symptoms and emphasizes the need for ongoing research into the long-term neurological sequelae of SARS-CoV-2 infection. These efforts are vital for improving our understanding of potential connections between viral infections and neurodegenerative disorders, ultimately leading to better diagnostic and therapeutic strategies for conditions such as CJD. This case report documents an instance of CJD onset in a patient with a recent COVID-19 infection. While the temporal association is notable, no causal relationship can be established. Further research is needed to explore potential links between viral infections and prion diseases, and clinicians should remain vigilant for unusual neurological presentations in patients with a history of COVID-19.

## Figures and Tables

**Table 1:** Timeline of Creutzfeldt–Jakob disease case following COVID-19.

Date	Event
October 2022	Diagnosis of COVID-19 with mild symptoms (dry cough, fatigue, shortness of breath); resolved in 7–10 days
Late November–early December 2022	Onset of speech difficulties (stuttering, foreign accent syndrome)
January 2023	Initial brain MRI showing diffusion restriction in bilateral occipital, parietal, and frontal lobes. Initial EEG normal
February–March 2023	Neurology evaluation; speech difficulties worsened. Lumbar puncture and EEG unremarkable. Treated with clonazepam and gabapentin
April 2023	Methylprednisolone infusion (1 g/day for 5 days); no improvement. Repeat MRI showed similar findings
May 2023	Hospitalization for progressive neurocognitive decline (catatonia, aggression, delusions). MRI showed progression of cortical diffusion restriction to temporal lobes. CSF analysis showed elevated tau and 14-3-3 proteins; RT-QuIC was initially negative. EEG showed generalized periodic discharges and ictal-interictal continuum patterns
June 2, 2023	RT-QuIC test returned positive, confirming CJD diagnosis. Decision to transition to hospice care
July 15, 2023	Transferred to hospice care due to rapid decline in cognitive, speech, and physical function
September 2023	Patient's demise (within 1 year of symptom onset, approximately October 2022–November 2023)

## Data Availability

Data sharing is not applicable to this article as no datasets were generated or analyzed during the current study. The data that support the findings of this study are available from the corresponding author upon reasonable request, subject to restrictions due to patient confidentiality and ethical considerations.
